# Bibliographical

**Published:** 1872-07

**Authors:** 


### BIBLIOGRAPHICAL.

A HAND-BOOK OF OPERATIVE SURGERY. By John H. Packard, M. D., one of the Sugeons to the Episcopal Hospital, etc., with fiifty-four steel plates and numerous illustrations on wood. Philadelphia: J. B. Lippincott & Co., 1870. Through Phillips & Crew, Atlanta, Ga.

The author has rendered valuable service to the profession by his hand-book—one that will be prized by students and practitioners. Having the merit of conciseness, it still brings before the eye, by well-executed illustrations, "one good method, practically described, for every surgical operation in general use at the present day." We know of no similar work so fully adapted to the practical, every day wants of the practitioner—one so copiously and accurately illustrated, and so conveniently arranged for easy reference.

Botany of the Southern States—In two parts. Part I: Structural and Physiological Botany and Vegetable Products. Part II: Description of Southern Plants. Arranged on the Natural System. Preceded by a Linnæan and a Dichotomous Analysis. By Prof. John Darby, A. M. New York: A. S. Barnes & Co., 111 & 113 William Street. 1866. Through Phillips & Crew, Atlanta, Ga.

The work of Prof. Darby has received the unqualified endorsement of Southern Botanists. To the Southern physician who would know the value and extent of the medicinal plants of his native soil, the work of Prof. Darby is invaluable, and we cordially recommend it.

Fireside Science. A series of popular Scientific Essays upon Subjects Connected with Every-Day Life. By James R. Nichols, A. M., M. D., Author of "Chemistry of the Farm and the Sea," and Editor of the Boston Journal of Chemistry. New York. Published by Hurd & Houghton. Cambridge: Riverside Press. 1872. Price $1 50.

Dr. Nichols is an accomplished chemist and writer, and in the work before us he has given many valuable and interesting essays "upon subjects connected with every-day life." The volume is replete with facts drawn from nature, and written in the charming style of one thoroughly familiar with his subjects. It is a work admirably adapted to the wants of the young—furnishing delightful reading with "truth which is stranger than fiction."

General Surgical Pathology and Therapeutics. In Fifty Lectures. A Text-Book for Students and Physicians. By Dr. Theodor Billroth, Professor of Surgery in Vienna. Translated from the fourth German edition, with special permission of the Author, by Charles E. Hackley, A. M. M. D., Surgeon to the New York Eye and Ear Infirmary, etc. New York: Appleton & Co. 549 & 551 Broadway. 1871.

This valuable work has reached its fourth edition. It covers a wide field, and the subjects of which it treats must always command the attention of the scientific physician. It is generally conceded that the study of Pathological Anatomy has made greater strides in Germany than any other country, and Prof. Billroth stands among the first of his countrymen as distinguished in Surgical Pathology. The work should be studied by every student, and be in the library of every physician. It is illustrated by 152 wood cuts, which add much to its value. It is a work of great merit, and we cordially recommend it to the profession.

A Practical Treatise on the Diseases of Children. By Alfred Vogel, M. D., Professor of Clinical Medicine in the University of Dorpot, Russia. Translated and Edited by H. Raphael, M. D., Late House Surgion to Bellvue Hospital, etc. Second American from the fourth German Edition. Illustrated by Six Lithographic plates. New York: D. Appleton & Co. 1871.

The profession in this country are placed under great obligations to Dr. Raphael for his translation of Vogel's excellent treatise on the diseases of children. It is an invaluable text-book for the student and practitioner—one which, from its practical character, must commend it to every earnest physician. The translator remarks: "The facts of Vogel's 'Kinderkrankheiten' having been translated into three other languages, and of its having attained to the fourth edition in less than eight years, together with the flattering commendations of the critics in various countries, and his belief in its utility and merit, and its adaptation to the wants of the practitioner and the student, must account for the translator having undertaken to render an English version of it." For which he, undoubtedly, has the thanks of the profession of America.

A Treatise on the Diseases of the Bones. By Thomas M. Markoe, M. D., Professor of Surgery in the College of Physicians and Surgeons, Surgeon of the New York Hospital, etc. New York: D. Appleton & Co. 1872.

Diseases of the bones has been a life-long study with the author, and he has given his brethren a work full of interest and scientific value. It cannot fail to profit all who desire to know more of the diseases of which it specially treats—diseases, many of which constantly come under the observation and, demand the best scientific efforts of the practitioner for their removal or relief.

Medical Thermometry, and Human Temperature. By C. A. Wunderlich, Professor of Clinic at the University of Leipzig, etc., and Edward Seguin, M. D. New York. William Wood & Co. 27 Great Jones St. 1871.

The importance of medical thermometry cannot be too highly valued, and its study should engage the best efforts of every consciencious physician. The author has prepared a practical work of facts, gathered from observations earnestly pursued, through sixteen years. He asserts that the more his "observations multiplied the more firm" his "conviction was, of the value of this method of investigation." "A knowledge of the course of temperature in disease is indispensable to medical practitioners. Because: all the phenomena of the sick are deserving of study. The temperature may be determined with a nicety which is common to few other phenomena. The temperature can neither be feigned nor falsified. We may conclude the presence of some disturbance in the economy from the mere fact of altered temperature. Certain degrees indicate that there is fever. The height of the temperature often decides both the degree and the danger of the attack. Thermometric observation may aid in the discovery of the laws regulating the course of certain diseases. When once the normal course of certain diseases has been determined, thermometry is able to simplify and confirm the diagnosis. Thermometric investigations indicate rapidly and surely any deviation from the regular course of the disease. The behavior of the temperature during the progress of the disease discovers either relapses or ameliorations before they could be otherwise recognized. In this way thermometry is able to regulate therapeutics. It puts us on our guard against any injurious influenc that may affect the patients in the course of their illness. It serves to indicate the transition from one stage of the disease to another, and particularly the commencement of convalescence and its complete establishment. It reveals complications, and how far recovery is from being complete. It generally reveals the imminence of a fatal termination. It announces the impossibilty of the continuance of life."

